# The Effect of a Physiotherapy Intervention on Thoracolumbar Posture in Horses

**DOI:** 10.3390/ani10111977

**Published:** 2020-10-28

**Authors:** Amy Shakeshaft, Gillian Tabor

**Affiliations:** Equine Performance Research Centre, Hartpury University, Gloucester GL19 3BE, UK; amyshakeshaft66@gmail.com

**Keywords:** equine physiotherapy, equine rehabilitation, equine posture, objective measurement

## Abstract

**Simple Summary:**

Altered spinal posture in a horse often relates to an underlying musculoskeletal condition that results in a negative effect on performance and welfare. Equine physiotherapy aims to adapt a horse’s posture by prescribing strengthening exercises to improve spinal range of movement and facilitate contraction of specific muscles. The aim of this study was to test the reliability of an outcome measure to objectively measure thoracolumbar posture before and after physiotherapy exercises over a specific time-period. The adapted flexicurve ruler (aFCR) had excellent intra-rater reliability compared to a standard flexicurve ruler. There were significant increases in thoracolumbar flexion after physiotherapy intervention at the specific time periods, of thirty minutes and one hour, however no significant changes at twenty-four hours. The physiotherapy intervention tested was shown to produce a short-term postural change, and over time may allow a long-term postural change with frequent practice. Outcome measures are valuable within veterinary physiotherapy to support clinical reasoning and the aFCR is shown to measure thoracolumbar posture pre and post-physiotherapy. Creating a postural change in horses can aid in a rehabilitation programme with veterinary physiotherapy, aiming to relieve pain and stiffness.

**Abstract:**

Dynamic mobilisation exercises (DME) are often used as part of a physiotherapy rehabilitation programme. Whilst immediate kinematic effects have been measured, the change in posture is anecdotally reported to have a longer duration. This study aimed to test the reliability of a simple objective measurement method, suitable for use in clinical practice, and to objectively measure equine thoracolumbar posture, before and after DME. A single investigator took triplicate measurements of the sagittal thoracolumbar shape using a flexicurve ruler (FCR) then triplicate measurements of the thoracolumbar shape using an adapted FCR (aFCR) in 37 horses. Subsequently, the thoracolumbar shape of 12 horses was measured using the aFCR before random allocation into two groups. Six horses acted as a control group and six horses underwent a series of DME, which included cervical flexion and lateral flexion baited stretches. Measurements were repeated prior to DME, at thirty minutes, one hour and at twenty-four hours after DME to assess thoracolumbar posture. The aFCR ruler had excellent intra-rater reliability compared to a standard FCR (aFCR: *p* = 0.146; intraclass correlation coefficient (ICC) 0.971; FCR: *p* = 0.0001; ICC 0.979). Significant increases in flexion occurred in the thoracolumbar region at 30 min (*p* = 0.027) and one hour (*p* = 0.046) after DME, but not at 24 h (*p* > 0.05) with no significant differences in the control group (*p* > 0.05) between baseline and subsequent times. The results suggest DME create a short-term postural change, determined by using an aFCR, which supports their use as part of a veterinary physiotherapy rehabilitation programme.

## 1. Introduction

Equine spinal conditions have become recognised as important factors impacting poor performance in horses [1]. Horses that present with pain and muscle spasm in the thoracolumbar spine often present with an altered movement in walk and trot, as well as an increase in extension and stiffness of this region, which is considered an abnormal posture [2]. This change in posture may further contribute to bony changes and soft tissue injury as a result of increased spinal extension, often referred to as lordotic posture [3,4,5,6]. In man, an anterior pelvic tilt, often referred to as lumbar lordosis, is associated with a number of musculoskeletal conditions, including back pain and a loss of core stability [7]. Lordotic postures are reported to cause an increase in loading spinal structures [8], with a more neutral spinal posture suggested by clinicians to reduce back pain in man [9]. Maintaining a neutral spinal posture in horses could reduce risk of pain and muscle spasm by allowing more space between the dorsal spinous processes, reducing risk of impingement (kissing spines) [10]. To achieve a neutral spinal posture, the hypaxial and epaxial muscles should work in unity to maintain the position of the spine with a straight or slightly arched upwards position [11].

Within equine rehabilitation regimes, specific strengthening exercises are commonly prescribed to adapt a horse’s posture, allowing a facilitation of muscle contraction, with the aim of creating an improved range of spinal movement to relieve pain and stiffness [12]. Anecdotally these exercises are suggested to effect changes in thoracolumbar posture, however little empirical evidence is available to support this assumption. Previous studies have investigated the effect of dynamic mobilisation exercises (DME) on the epaxial muscle multifidus cross-section area, with positive effects reported over a three-month period [13,14]. Although increases in cross-sectional area of multifidus, stride length and intervertebral motion during DME have been reported [15,16], the effect of DME on posture over time has not been tested. 

Assessing a horse’s rehabilitation progress, with objective measurement methods, ensures the physiotherapist meets their duty of care [17]. A flexicurve ruler (FCR) is a strip of lead, covered in rubber, which can be moulded to the contours of the spine and is a measurement tool validated by Greve and Dyson [18] to assess the shape of the horse’s back. It is considered a useful tool during an assessment of transverse muscular profile [18], however, to provide an accurate representation of thoracolumbar posture and identify thoracolumbar lordosis, measurement in the sagittal plane is required. One challenge of adapting the FCR for use in the sagittal alignment, is potential error in reporting due to not keeping the relationship between the sagittal profile of the horse’s spine and the FCR horizontal, thus increasing or reducing the depth of the lordosis. Adopting a modified method for FCR use, by connecting the ruler to a spirit level to ensure the sagittal profile maintains in the same ratio to the horizontal plane of the horses back when recording posture, could counteract measurement error. If reliable, this adapted FCR (aFCR) could be used as an objective measure to record the outcome of a physiotherapy intervention, for instance the effect of DME, adding rationale to specific treatments. The aim of this study therefore was to test the reliability of the aFCR to objectively measure thoracolumbar posture, before and after DME in horses over a specific time period.

## 2. Materials and Methods 

Ethical approval was gained prior to data collection via the Ethics Committee at Hartpury University (ETHICS2018-01-LR.).

### 2.1. Part A: Testing Reliability of the aFCR

Thirty-seven horses of mixed age, breed, workload (13.5 ± 6.4 years) and height (153 cm ± 15.9 cm) were included in the study. To be eligible for inclusion, horses were required to have no current evidence of lameness, trauma or spinal surgery within the last year. An Association of Chartered Physiotherapists in Animal Therapy (ACPAT) Physiotherapist assessed the horses prior to data collection to assess suitability for the study. 

#### Measurement Protocol 

A single, experienced veterinary physiotherapist took all FCR and aFCR measurements of the horses’ spinal posture. The investigator was blinded to their own measurements by an additional investigator (ACPAT chartered physiotherapist), drawing and recording the measurements from the FCR and aFCR. Thoracolumbar posture was recorded in triplicate for each condition and time point. The measurements for both pieces of equipment were not randomised. 

All measurements were taken with the horse held by a handler, wearing a head collar, on a flat surface in their stable. Horses were stood “square” with their head and neck in a neutral position to minimise any lateral flexion prior to measurements [18]. The thoracic spinous processes of each horse were identified by manual palpation prior to the FCR and the ruler was aligned and shaped to the contour of the dorsal spinous processes, caudal to the highest point of the wither. The ruler was then removed and laid onto A3 paper with the cranial end of the FCR laid at the start of a 30 cm pre-drawn straight line on the paper and traced the outline of ruler. At 5cm intervals along the FCR, vertical lines were drawn to meet the horizontal line to assess the curvature change, measured (mm) by the second physiotherapist and recorded for analysis.

**Figure 1:**
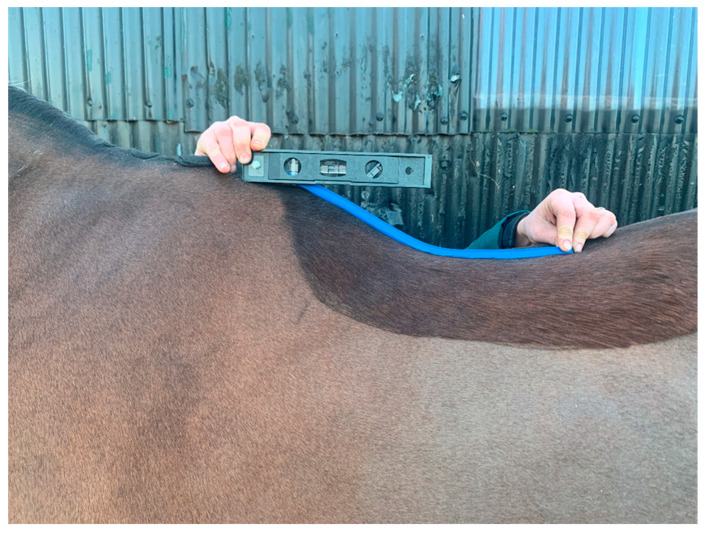
The adapted flexicurve ruler placed on the horse’s spine in a sagittal plane to measure thoracolumbar shape. The spirit level is aligned to horizontal and butterfly nut tightened to retain the flexicurve’s orientation to the horizontal, before removal to trace sagittal profile.

**Figure 2:**
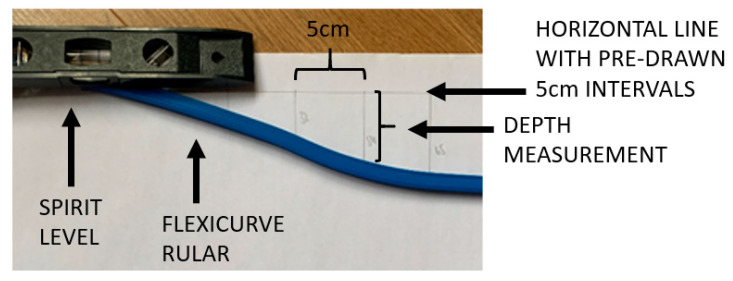
The aFCR (adapted Flexicurve Ruler), with the spirit level parallel to the pre-drawn 30 cm horizontal line. The horizontal line is split into intervals of 5 cm (5 cm, 10 cm, 15 cm, 20 cm, 25 cm, 30 cm). The distance between horizontal line, at these marked intervals, and the trace of the sagittal profile was measured in millimetres.

Within the aFCR method, the ruler was attached to a spirit level with a bolt and butterfly nut, which was tightened when the spirit level was rotated to align horizontal after the FCR had been aligned and shaped to the contour of the thoracic spinous processes (Figure 1). The spirit level was aligned with the pre-drawn straight line and the outline of the FCR was traced below the pre-drawn horizontal line to record the shape of the dorsal thoracolumbar posture (Figure 2). 

### 2.2. Part B: Measurement of Posture Before and After DME

A further twelve horses of mixed breed, age (13 ± 6.3 years) and height (160 cm ± 13.4 cm) were recruited through convenience sampling for part B of the study and were subject to the same exclusion criteria as for Part A. Horses were randomly allocated to either the control group (*n* = 6) or intervention group (*n* = 6). Both groups had a pre-testing measurement taken using the aFCR protocol and the same investigator in Part A. 

Horses in the intervention group then performed a programme of six DMEs applied by an ACPAT physiotherapist: chin-to-chest, chin-between-carpi, chin-to-fore fetlocks, chin-to-girth, chin-to-flank and chin-to-tarsus exercises and each exercise was performed to the left and right side for the lateral exercises. Each mobilisation was repeated five times during an exercise session and each position held for five seconds [13]. Carrots were used as bait for the exercises, to motivate the horse to perform each exercise, whilst in their own stables with their own bedding. The exercise was completed when the horses’ nose reached the anatomical position they were then asked to stretch to and hold the position for five seconds.

The control group did not receive any DME and remained in their own stable for the same duration as the intervention group whilst the DME were carried out. All horses remained on their same individual turnout or stabling routine but did not undergo any other exercise within the 24 h of testing.

Thoracolumbar posture of both groups was measured using the aFCR protocol prior to DME at 0 min and at 30 min, 1 h and 24 h after DME in the intervention group or the control period in the second group. The measurement for each time interval was taken once due to the repeatability of the aFCR found in Part A. 

### 2.3. Data Analysis 

The measurements (mm) from Part A and Part B were recorded in Microsoft Office Excel (Microsoft Excel for Mac 2011, version 14.4.4, Redmond, WA, USA). Data met non-parametric assumptions due to small sample size and mixed distribution [19] and SPSS Statistics (IBM SPSS Statistics for Mac, version 26, Chicago, IL, USA) was used for analyses.

Part A: Repeated measurement data were analysed using a two-way mixed intraclass correlation coefficient (ICC) to test the intra-rater reliability [20]. The 95% confidence intervals (CI) were calculated based on a mean rating of k = 3. The ICC value was interpreted to assess the reliability (0.0–0.5—poor, 0.5–0.75—moderate, 0.75–0.9—good, 0.9 and above—excellent) [21]. Friedman’s analyses established if differences occurred in FCR and aFCR values between the three measurements recorded. Significance was set at *p* < 0.05 

Part B: A series of Freidman’s analyses, with significance set at *p* < 0.05, with post hoc Wilcoxon signed-rank tests assessed if changes in TL posture occurred over the four different time periods for each group. The measurements at each level (5 cm, 10 cm, 15 cm, 20 cm, 25 cm and 30 cm) were summed to obtain a total value of the thoracolumbar shape. 

## 3. Results

### 3.1. Part A: Intra-Rater Reliability 

No significant differences were found between the three aFCR measurements taken (*n* = 37) at any of the six levels recorded: 5 cm, 10 cm, 15 cm, 20 cm, 25 cm and 30 cm (*p* > 0.05). This method was found to have excellent intra-rater reliability (χ2(2) = 3.854, *p* = 0.146; ICC: 0.971; CIs: 0.964–0.977). For the standard FCR method, significant differences occurred between the three measurements (χ2 (2) = 23.01, *p* = 0.0001; ICC: 0.979; CIs: 0.976–0.983) (Koo and Li, 2016). The adapted FCR was therefore taken forward to use as a reliable measure in part B of this study.

### 3.2. Part B: Measurement of Posture before and after DME

**Figure 3:**
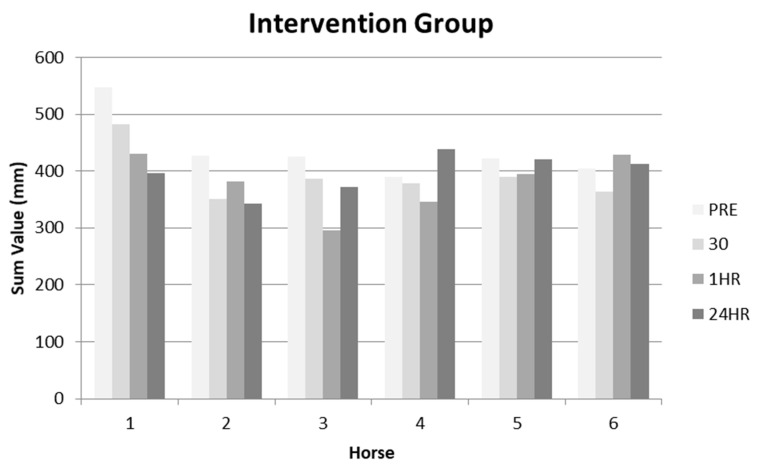
Summed data for the intervention group at time periods: before (PRE), thirty minutes, one hour and twenty-four hours after DME.

**Figure 4:**
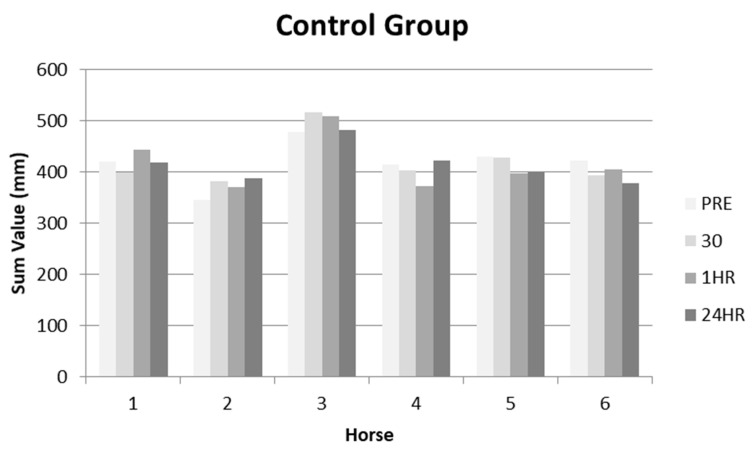
Summed data for the control group at time periods: before (PRE), thirty minutes, one hour and twenty-four hours after the intervention groups DME.

The measurements for each horse, at each time period taken from the aFCR were summed to assess cumulative thoracolumbar posture change for both the intervention and control group (Table 1). The reduced values were considered to equate to a reduction in lordotic posture (Figure 3 and Figure 4). 

No significant differences in measurements were found between the time intervals (χ2(2) = 0.333, *p* = 0.846) in the control group. In contrast, significant differences in thoracolumbar posture were reported for the DME group over the four time intervals, showing a decrease in lordosis (χ2(2) = 6.333, *p* = 0.042). Subsequent post hoc analysis identified horses presented with a reduced lordotic posture for 30 min (*p* = 0.027) and 1 h (*p* = 0.046) when compared to the pre-DME measurement. This effect, however, was not evident 24 h after the initial pre-DME measurement with horses recording an increase in lordotic posture from 30 min and 60 min, however this was not found to be significant (*p* > 0.05). Within the control group, no significant differences were found in thoracolumbar posture between pre and post DME measurements (*p* > 0.05).

## 4. Discussion

The aim of this study was to test the reliability of an outcome measure to investigate thoracolumbar shape in horses in the sagittal plane and to assess if changes in this measure of equine thoracolumbar spinal posture occurred after completion of DME. The reliability of the aFCR was tested with 37 horses and a further 12 horses were then used to assess whether DME created a postural change over certain time periods. DME created significant short-term posture change within the intervention group, and the control group did not achieve any postural change during the same time period. 

### 4.1. Reliability of the aFCR

Measurement of thoracolumbar shape was found to be repeatable for one rater when using the aFCR with excellent reliability for the majority of the results [21], supporting use of this piece of equipment within equine physiotherapy and rehabilitation clinical practice. The excellent reliability reported suggests the equipment will allow physiotherapists to measure spinal posture in horses throughout the treatment process to assess any improvements. 

### 4.2. Assessing Long-Term Postural Change

The objective for the second part of this study was to assess whether the aFCR ruler should objectively measure any postural change over 24 h as a result of a physiotherapy intervention. No significant changes occurred within the control group when comparing pre-DME measurement and post-DME measurements taken at thirty minutes, one-hour and twenty-four hours. Interestingly, for the intervention group, significant changes were found for thoracolumbar posture. These were a 10.1% (cohort mean) reduction in measurements between pre-DME vs. post-DME at thirty minutes, and a 13.0% reduction in measurements post-DME at one hour; with the results demonstrating the intervention induced a more flexed thoracolumbar posture. There were no significant results after 24 h, suggesting that the intervention facilitates a transient effect on thoracolumbar posture with a subsequent return to an increase in lordosis. Although not statistically significant at 24 h the mean percentage difference from baseline for the intervention group was still 9.0% less that the baseline measurements 

Horses that perform DME over longer periods have been shown to have significant increases in multifidus cross-section, which potentially could reduce instability of the intervertebral joints during gait and thus spinal degenerative changes [13,14] and although these previous studies have measured over a three-month period, the DME exercises effected a postural change within the short time frame of this study. Ten DME were carried out for each horse, repeated five times and held for five seconds, which require activation of the epaxial and hypaxial muscles, creating flexion through the thoracolumbar spine [15,16], and creating the change in thoracolumbar posture which lasted beyond the duration of the exercises. 

A standard veterinary physiotherapy session will usually last around one hour, consisting of a subjective and objective assessment; manual techniques and exercises to reduce back pain and elicit a postural change [22]. Establishing that a postural change can be measured within one hour of treatment supports the rationale for use of DME and objective measurement within the physiotherapy session. Frequent practice of the DME for an owner would allow the exercise to establish a long-term postural change. Owners are routinely given home exercises to carry out with their horse’s, such as DME, and repeating these exercises will elicit a muscular and postural change over a longer period of time potentially improving measurements in future sessions. In previous research, it has been suggested that DME should be carried out between three to five times per week, continuing the same baited stretches programme [13,14]. In this study, the change in posture was evident after one hour but not at 24 h, suggesting a daily exercise frequency is required, if change in posture is a targeted outcome, however further time periods, such as twice a day—morning and evening—could be investigated.

When assessing spinal kinematics, Gomez Alvarez et al. [23] concluded that elevated head and neck positions induce extension in the cranial thoracolumbar spine and create some flexion in the caudal of the thoracolumbar spine. The pre vs. twenty-four hours measurement produced no significant changes, meaning the posture returned close to the pre-measurement lordotic thoracolumbar position. Horses were kept in their stables between measurements, with no haynet or feed for the 30 min and 1 h measurements, due to the time of day the study was carried out. Between 1 h and 24 h measurements, horses had their usual overnight stabling and feeding routine. All horses were given a haynet overnight, placed high on the stable wall and tied to a haynet ring, which may substantiate the change in thoracolumbar posture when measured at the 24 h point, the day after the horses were fed from a haynet overnight. 

### 4.3. Study Limitations

The aFCR has shown to be a reliable objective tool to assess spinal posture in horse’s which allows clinicians to make an informed treatment decisions regarding horse’s rehabilitation plan, however the study did present with some limitations. There was no blinding of the physiotherapists to the grouping of the horses in part B of the study which may have introduced bias, however, the single researcher was experienced in using the aFCR. Additionally, due to the amount of horses recruited and time restraints, only a small sample size was used in this part of the study (*n* = 12). Small sample studies can affect the statistical power, affecting the likelihood that a statistically significant finding reflects a true effect [24]. Although the sample size is small, previous studies have used a similar or smaller sample size, gaining significant results [25,26]. During the testing period, between 1 h and 24 h, the horses were provided with their usual feeding requirements over night to meet the needs of basic care, however this allowed them to change their movement and posture without the control of the physiotherapist. 

### 4.4. Future Research

Assessing more time periods would allow clarification to determine when changes occur and assessing the cumulative effect between the treatments across a number of days would be valuable. It would be noteworthy to see if results differed at twenty-four hours for the intervention group if the horses grazed from the floor overnight instead of using a haynet, reducing an elevated head and neck posture creating flexion of the thoracolumbar spine and increasing the intervertebral distances between the adjacent thoracic dorsal spinous processes [10]. It could be suggested that horses with thoracolumbar extension would benefit from grazing from the floor, allowing flexion of the spine and reducing a continuous extended posture that may be caused by eating from haynet. 

## 5. Conclusions

A lordotic thoracolumbar spine posture has been related to conditions such as over-riding dorsal spinous processes, osteoarthritis and muscular pain, which affect performance. Early recognition of an altered posture or pain ensures that treatment can begin, reducing the chance of developing performance problems in their future career. Within physiotherapy, outcome measures are valuable for the clinical reasoning of treatments. Intra-rater reliability of the aFCR was found to be excellent as an inexpensive, non-invasive, easy to use outcome measure it is ideal to use to measure thoracolumbar posture. This could especially be useful in pre and post-physiotherapy treatment to assess posture changes and effectiveness of specific interventions as well as rehabilitation programmes. The DME have shown to produce a postural change within a specific time period, and with frequent practice will allow a long-term postural change in horses, enabling a decrease in lordotic posture. 

## Figures and Tables

**Table 1:** Individual horse summed measurements in millimetres (mm) at time periods: before (PRE), thirty minutes, one hour and twenty-four hours after dynamic mobilisation exercises (DME).

Group	Horse	PRE (mm)	30 min (mm)	1 h (mm)	24 h (mm)
Intervention	1	547	482	430	396
	2	427	351	382	343
	3	426	386	296	372
	4	390	379	346	438
	5	423	390	394	421
	6	404	364	428	412
Control	7	421	399	444	418
	8	345	383	371	387
	9	479	516	509	482
	10	415	404	372	422
	11	430	428	398	399
	12	422	394	405	379
